# Endobiliary Radiofrequency Ablation for Hepato-Biliary Diseases: A Narrative Review

**DOI:** 10.3390/diseases13080273

**Published:** 2025-08-21

**Authors:** Tawfik Khoury, Wisam Sbeit, Andrea Lisotti, Bertrand Napoléon

**Affiliations:** 1 Department of Gastroenterology, Galilee Medical Center, Nahariya 22100, Israel; wisams@gmc.gov.il; 2 Faculty of Medicine in the Galilee, Bar-Ilan University, Safed 5290002, Israel; 3 Gastroenterology Unit, Hospital of Imola, University of Bologna, 33, 40126 Bologna, Italy; lisotti.andrea@gmail.com; 4 Department of Gastroenterology, Hôpital Privé Jean Mermoz, Ramsay Santé, 69008 Lyon, France; dr.napoleon@wanadoo.fr

**Keywords:** RFA, biliary, malignant, obstruction, safety, efficacy

## Abstract

Background/Objectives: Malignant biliary diseases still represent a therapeutic challenge given its poor prognosis, and limited response to the available curative treatments. Recently, endobiliary radiofrequency ablation (RFA) has been increasingly reported as an adjunct therapeutic option for biliary diseases, especially malignant biliary obstruction (MBO), due to potentially improving survival and stent patency. Methods: Herein, we provide a comprehensive review article discussing the indication, procedural details, safety, and comparative efficacy of endobiliary RFA to aid in providing an in-depth understanding of the clinical indications and future implications of this specific option. Results: Overall, endobiliary RFA is technically feasible, being associated with a high safety profile, significantly improving biliary stent patency, and having a potential benefit in extending the survival of patients with MBO who were treated with endobiliary RFA combined with biliary stenting vs. stenting alone. Moreover, it has a promising role in the treatment of intraductal extension of ampullary tumors. Conclusions: Endobiliary RFA had a beneficial therapeutic effect in biliary strictures, with potential impact on patients outcome and survival.

## 1. Introduction

**Figure 1:**
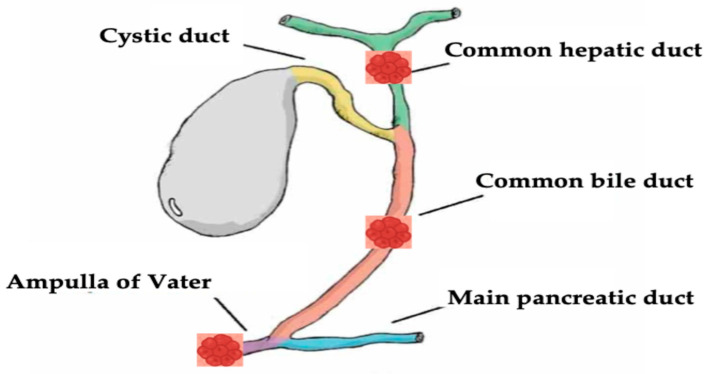
Neoplastic hepato-biliary sites of endobiliary RFA discussed in this review.

Biliary tract diseases, specifically malignant biliary diseases such as cholangiocarcinoma, are still associated with poor prognosis with a high mortality rate [1,2,3]. Traditionally, surgical resection is the main definitive treatment option that was shown to have a good outcome in appropriately selected patients [4]. However most of the patients show an advanced disease stage at presentation, and the treatment aim is palliative with systemic chemotherapy and radiotherapy [5,6,7]. Even with systemic therapy, patients still do not survive more than 1 year [8]. Given the dismal prognosis and the limited efficacy of systemic chemoradiotherapy, local ablative therapies have been evaluated and used in biliary diseases, as they aimed to induce tissue apoptosis at the application locations, including irreversible electroporation [9], microwave ablation [10,11], ethanol application, cryoablation [12], and photodynamic therapy [13,14]. However, they were associated with limited efficacy [15,16]. Recently, endoscopic guided radiofrequency ablation (RFA) has been increasingly used in the setting of biliary disease, especially in MBO. Previous studies showed that RFA improves both biliary and hepatic tumor prognosis [17,18]. Moreover, endobiliary RFA should be a curative therapeutic option among patients with hepatic and biliary malignancies [19,20,21]. The purpose of this article is to provide an in-depth review of the evolving role of RFA in the management of hepato-biliary diseases, focusing on both malignant (Figure 1), and select benign indications.

## 2. Mechanism of Action

RFA aims to provide a high thermic alternating current to induce coagulative necrosis. The thermal waves cause ionic perturbation in the tumoral tissues, leading to a high temperature ranging from 60 °C and 100 °C from ionic friction, resulting in protein denaturation and irreversible cell death [22,23].

RFA is performed via inserting RF electrodes into the tumoral tissue to administer RF potential current, leading to cell destruction via the generation of high temperatures within the tumor environment [24]. The process of cell destruction occurs through the Joule effect [25,26], which is the friction of the ions in the tumorous tissue secondary to high-frequency alternating current (400–500 kHz) [27,28], leading to high thermal injury, protein denaturation, melting of the lipid bilayer, and cell death [29].

**Figure 2:**
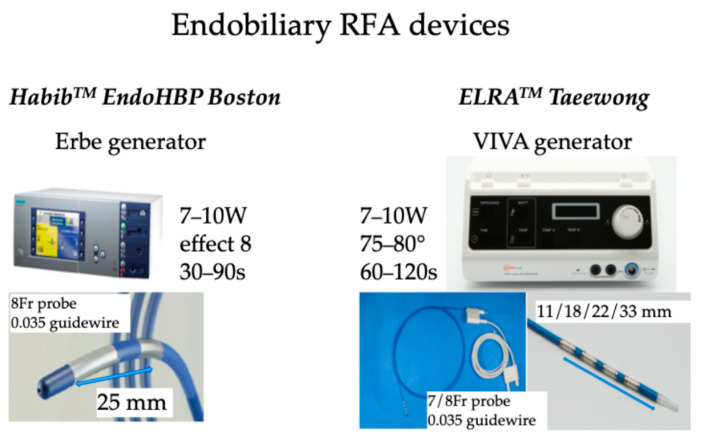
RFA devices used for endobiliary RFA.

Additionally, RFA induces local damage confined to 2–5 mm depth, thus limiting the injury of surrounding normal tissue [30,31,32]. There are several routes to administer RFA, including percutaneous [33,34], surgical [35], and recently endobiliary-guided RFA [36,37]. In endobiliary RFA applications, two companies provide probes: the Habib™ EndoHPB (Boston Scientific, Marlborough, MA, USA) catheter is an 8 Fr device of 1800 mm long with two electrodes at the distal tip of the catheter with an 8 mm space between the two electrodes; ELRA^TM.^(STARmed, Goyang, Republic of Korea) proposed 7 or 8 Fr bipolar catheters of 1750 mm length with two to four electrodes in a distal segment of variable lengths (11, 18, 22, and 33 mm) with a depth of circumferential ablation of 6–8 mm and a median ablation depth of 4.0 mm [38] to facilitate RFA of pathologies of variable lengths [39]. An automatic temperature setting is provided by the specific generator to avoid exaggerated temperature application, thus limiting tissue damage to the surrounding normal tissue [40,41,42]. The recommended setting is to use 7–10 Watts power with a defined temperature of 80 °C for up to 2 min [43,44]. The probe is introduced in the operative channel of a duodenoscope during the endoscopic retrograde cholangiopancreatography (ERCP) procedure. Post-ablation, a metal stent is placed to maintain ductal patency. These catheters deliver circumferential current to the biliary wall using monopolar or bipolar modes, aimed at tumor debulking and improving biliary stent patency during ERCP; after cannulating the common bile duct, a guidewire is introduced through the stricture, and then the RFA catheter is placed across the stricture under fluoroscopic guidance, and RFA current is applied for almost 2 min. Endobiliary RFA can also be applied to manage tumoral tissue ingrowth into the metallic stents placed for palliation of obstructive jaundice (Figure 2).

## 3. Indications of Endobiliary RFA in Malignant Disease

### 3.1. Malignant Hilar and Extrahepatic Biliary Stricture

Currently, the most widely used indication of endobiliary RFA is for the treatment of unresectable malignant biliary stricture, as it is used in conjunction with biliary metallic stents to hinder tumoral ingrowth [45]. MBO can be intrinsic mainly due to cholangiocarcinoma (CCA) or secondary to extrinsic malignancies such as pancreatic adenocarcinoma [46]. CCA accounted for approximately 2–3% of all gastrointestinal malignancies, as it is the most common primary hepato-biliary tumors [47,48]. CCA can be located in the extrahepatic or intrahepatic bile ducts according to their ductal extension and relation to the cystic duct according to the Bismuth–Corlette system [49]. The mainstay treatment for CCA is still surgical resection when the tumor is eligible for resection [50]; however, in cases of unresectable CCA that causes biliary obstruction, chemoradiotherapy (including immunotherapy) is the usual treatment accompanied by biliary metallic stent placement to relieve biliary obstruction [51]. However, these treatment modalities are associated with limited survival benefit, and the risk of biliary stent ingrowth by the tumoral tissue still not negligible. Therefore, several studies have assessed the role of endobiliary RFA on patient outcomes and stent patency rate. The initial experience with cohort studies only reported the safety of RFA. Steel et al. reported the safety of endobiliary RFA in 22 patients with unresectable malignant biliary obstruction and showed a technical success of 95.5% and adverse event rate of 13.6% (1 patient developed acute pancreatitis, and 2 patients had cholecystitis), without serious adverse events [43]. Another previous study by Figueroa-Barojas et al. reported 20 patients with unresectable MBO, where they treated 25 strictures, reporting a significant increase in the stricture luminal diameter from 1.7 mm (SD = 0.9 mm; range = 0.5–3.4 mm) to 5.2 mm (SD = 2 mm; range = 2.6–9 mm) after endobiliary RFA, with two cases of mild acute pancreatitis and acute cholecystitis [52]. Additionally, Dolak et al. reported 58 patients with MBO, mainly CCA, in 48 patients who underwent 84 RFA sessions, reporting 100% technical success, median stent patency of 170 days, and median survival of 10.6 months. Notably, 12 adverse events (14.3%) were reported (partial liver infarction in 1 patient, cholangitis in 7 patients, hemobilia in 2 patients, hepatic coma in 1 patient, and left bundle branch block in 1 patient) [53]. The largest cohort was reported by Sharaiha et al., who reported 69 patients with MBO, with excellent technical success and median survival of 11.46 months. Of note, adverse events occurred in seven patients (10%) including (one case of pancreatitis, two cases of cholecystitis, one case of hemobilia, and three cases of abdominal pain) [36]. Additional studies showed highly safe procedures without significant adverse events [54]. A previous study by Lee et al. including 30 patients (19 with cholangiocarcinoma, 9 with pancreatic cancer, and 2 with gallbladder cancer) reported a 100% technical success, stent patency rate of 236 days, and overall survival of 383 days. Notably, adverse events occurred in 10% of cases, mostly mild in severity [55]. Table 1 demonstrates the cohort studies on endobiliary RFA in malignant biliary obstruction.

Moreover, recent studies on endobiliary RFA used with a biliary metallic stent compared to a stent alone showed conflicting results. Sharaiha et al. reported a similar median survival of 5.9 months in both groups, with a similar stent patency rate and adverse event rate [41]. On the other hand, several recent studies have showed a beneficial effect of endobiliary RFA when added to regular biliary stenting. A retrospective case-controlled study by Kallis et al. including 69 patients (23 patients had RFA and stenting vs. stenting alone in 46 patients) showed a significant survival benefit in the combination group (226 days) vs. 123.5 days in the stenting group only (*p* < 0.01), with minimal adverse events and with similar stent patency rates (*p* = 0.67) [56]. Another study by Liang et al. showed a significant increase in the median survival and stent patency rate in the RFA + stenting group (*p* = 0.036, and *p* = 0.024, respectively) [57]. Similarly, a previous randomized controlled trial by Yang et al. including 65 patients with CCA, among them 32 patients who had RFA + stenting vs. 33 patients who had stenting only, showed a statistically significant increase in overall survival and in the stent patency rate (*p* < 0.001, and *p* = 0.02), respectively [21]. Moreover, a recent meta-analysis by Sofi et al. including nine papers of additional RFA to biliary stenting in MBO showed a significant increase in survival in the RFA group (95%CI: 1.145–1.7; *p* < 0.01), in addition to prolongation of stent patency [20]. A recent study published in this field showed encouraging results in term of efficacy and safety. Shin et al. conducted a propensity score-matched analysis including 32 patients assigned to endobiliary RFA + stenting vs. 32 patients with stent only. The technical success was 100% in both groups. The clinical success rate was 93.8% in the RFA + stent group and 87.5% in the stent-only group *(p =* 0.67). The median time to recurrent biliary obstruction was significantly higher in the RFA + stent group vs. the stent group only (242 vs. 168 days, respectively, *p* = 0.031). Notably, there was a trend toward higher overall survival in the RFA + stent group (337 days) as compared to 296 days in the stent group only (*p* = 0.26), while there was no difference in the rate of adverse events (12.5% vs. 9.4%), respectively [58]. On the other hand, Kang et al. reported a single-center prospective, randomized phase II TRIAL including 48 patients (24 patients in the RFA + stent group vs. 24 patients in the stent group only). The technical success rate was 100% in both groups, with similar clinical success of 87.5% vs. 83.3% in the RFA + stent vs. stent only groups (*p* = 1). Moreover, there was no difference in the 90-day stent patency rate and overall survival in both groups (58.3% vs. 45.8%, *p* = 0.39, and 244 days vs. 180 days, *p* = 0.28), respectively. Moreover, procedure-related complications (early ≤ 7 days) occurred in 4.2% in the RFA + stent group vs. 12.5% in the stent group only [59]. Table 2 demonstrates case-controlled studies. Finally, the addition of endobiliary RFA to stenting is a promising and encouraging therapeutic option in the management of malignant biliary obstruction to both improve patients’ overall survival and stent patency rate.

### 3.2. Biliary Intraductal Papillary Mucinous Neoplasm

Biliary intraductal papillary mucinous neoplasm (B-IPMN) is a rare tumor of the biliary tree defined by multiple adenomas characterized by papillary or villous neoplasms secondary to proliferation of the bile duct epithelium that can affect both the extrahepatic and intrahepatic bile ducts [61,62]. The World Health Organization (WHO) in 2010 recommendation to classify B-IPNB as a separate entity of neoplastic biliary diseases, accounting for 10% of all bile duct tumors overall [63].

Given that B-IPMN is a premalignant disease with high potential of malignant transformation of approximately 41% [64,65], surgical resection is still the only treatment for B-IPMN, including hepatic resection and liver transplantation for intrahepatic lesions. However, to date, only several case reports have been published on the use of endobiliary RFA for B-IPMN. Natov et al. reported a 76-year-old male patient with B-IPMN of the pancreato-biliary sub-type who underwent two sessions of endobiliary RFA, without evidence of recurrence at 10-months follow-up [66]. Delaney et al. reported 87-year-old female patients with left intrahepatic B-IPMN with low-grade dysplasia who underwent three sessions of endobiliary RFA a few months apart, with magnetic resonance imaging (MRI) performed 1 year after the last RFA showed complete resolution of the B-IPMN, without procedure-related adverse events [67]. Another case by Tang et al. showed a compete B-IPMN resolution at 8-months follow-up following the procedure, without any reported adverse events [68]. Although that the data are still emerging, endobiliary RFA could represent an alternative effective and safe therapeutic option for patients with B-IPMN who are not candidates for surgical intervention. Additional large studies with longer follow-up periods are warranted to determine the exact efficacy of endobiliary RFA for B-IPMN.

### 3.3. Intraductal Extension of Ampullary Tumors

**Figure 3:**
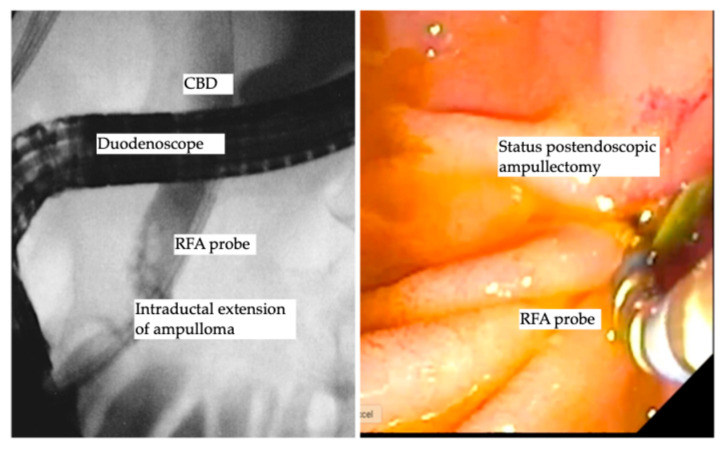
Endobiliary RFA for intraductal extension of ampullary adenoma.

Benign ampullary tumors are increasingly encountered in daily clinical practice. The cornerstone treatment is still endoscopic ampullectomy using an endoscopic snare, which is the standard treatment of choice, as the complete resection rate using endoscopic ampullectomy ranges from 67% to 92% [69,70,71]. The aim of complete R0 resection is not always achieved and further decreases in the cases of ampullary lesion extension into the distal bile duct [72]. Basically, the traditional treatment of intraductal extension was and is still the Whipple procedure (pancreaticoduodenectomy) [69,73], despite the high perioperative morbidity and mortality that might reach to 9% [74,75]. The data in this field are still scarce, as most of the data are based on case reports [76,77,78]. However, a few recent larger cohort studies reporting the role of endobiliary RFA showed promising results. Rustagi et al. reported a multicenter retrospective analysis of 14 patients with ampullary neoplasms extended into the common bile duct and 3 patients with main pancreatic duct extension, where technical success was reported as 100%, and treatment success, defined as the absence of neoplasms by intraductal biopsy, was achieved in 92% of patients. Six patients (42.8%) developed procedure-related adverse events, including five patients with ductal strictures and one patient with a retro-duodenal abscess, all of whom were successfully treated endoscopically [79]. Another prospective multicenter study by Camus et al. included 20 patients after endoscopic ampullectomy for ampullary tumors, with histologically proven remnant adenoma in the bile duct (ductal extent < 20 mm), where one session of endobiliary RFA using a power setting of 8 Watts for 30 s showed a residual neoplasia of 15%, and 30% at 6 and 12 months following endobiliary RFA, with an adverse event rate of 40%, while no major adverse events were reported [80]. A recent study used a novel temperature-controlled RFA probe device to decrease the rate of procedural-related adverse events, reporting ten patients who underwent RFA using the new (ELRA™) device and showed an adverse event rate of 30% (three patients); two of them developed mild pancreatitis, and one patient had asymptomatic biliary stricture. Notably, 90% of patients had undetectable adenomatous lesions over a median period of 253 days [81]. A recent study by Tringali et al. reported a technical success of 100% and clinical success of being recurrence free of 67% at a median follow-up of 21 months [82]. A recent study by Dahel et al. included 25 patients who underwent 40 sessions of endobiliary RFA for residual and recurrent neoplasia after endoscopic ampullectomy, showing a 100% technical success rate and clinical success rate of 90% (22/24 patients). Of note, the adverse event rate was 56%, as 2 cases were defined as early adverse events (1 case of acute pancreatitis and 1 case of bleeding) and 12 cases of late adverse events of biliary strictures that were managed endoscopically. Importantly, one patient died, for whom the pancreatic stent insertion failed due to acute severe pancreatitis post-endobiliary RFA, yielding a mortality rate of 4% [83]. Finally, endobiliary RFA for ductal extension of ampullary tumors carries a spark of hope with accumulating promising data that in the future may turn out to be the favorite therapeutic option for intraductal extension of ampullary tumors. Table 3 demonstrates the studies reported endobiliary RFA for intraductal extension of ampullary tumors. Figure 3 shows endobiliary RFA for intraductal extension of ampullary adenoma.

### 3.4. Malignant Ingrowth of Biliary Stents

The indications of endobiliary RFA have been extended, as recent studies reported the efficacy of endobiliary RFA for occluded biliary self-expandable metal stents (SEMSs) of tumorous tissue that showed conflicting results.

Kadayifci et al. reported a retrospective study including 25 patients with malignant occlusion of SEMSs who were treated with endobiliary RFA using the Habib™ RFA probe who were compared to 25 patients with an occluded biliary SEMS treated by stent placement. The technical success rate in the RFA group was 56% (14/25 patients); the remaining 11 patients who failed RFA were treated with stenting. The stent patency rate at 3 months was 56% in the RFA group compared to 24% in the stent-only group (*p* = 0.04). The median time of stent patency was 119.5 days vs. 65.3 days in the RFA and stent-only groups, respectively (*p* = 0.03). The adverse event rates were similar, without differences in the rate of survival at 30 days and 3 and 6 months survival [84]. Nayar et al. reported seven patients who underwent nine endobiliary RFA procedures using the ERLA™ probe. The mean stricture diameter was 1.13 ± 0.54 mm before RFA and 4.42 ± 1.54 mm after the procedure (*p* < 0.0001). Notably, 71% of patients required additional stenting to optimize biliary drainage. None of the patient developed procedure-related adverse events [85].

Still, the role of RFA in malignant ingrowth of SEMSs is still very scarce; however data are needed to better define its efficacy and to know whether endobiliary RFA of an occluded SEMS produces any additional effect on the tumor beyond the lumen of the stent.

## 4. Indications of Endobiliary RFA Benign Biliary Strictures

Benign diseases can also cause biliary strictures, such as intragenic injury secondary to surgical procedures, inflammatory processes, and post-liver transplantation [86,87]. The cornerstone therapy for benign biliary strictures is endoscopic insertion of multiple plastic biliary stents or biliary fully covered metallic stents, which is associated with high efficacy in resolving biliary strictures [88,89,90]. However, some strictures did not respond to endoscopic treatment. Therefore, few studies have assessed the efficacy of endobiliary RFA for benign biliary strictures. A previous study by Hu et al. reported nine patients with benign biliary strictures (three after liver transplantation, four post-surgery, and two inflammatory strictures) who underwent bipolar endobiliary RFA using a power setting of 10 Watts for 90 s. All patients had immediate improvement in the biliary stricture. At median follow-up of 12.6 months, four patients (44.4%) had complete stricture resolution without the need of further stenting, one patient had recurrence, one patient underwent surgery, and one patient died due to irrelevant biliary cause. Only one patient (11.1%) developed post-ERCP pancreatitis [91]. Another previous study reported a complete resolution in 10 out of 18 patients (55.5%) who underwent percutaneous transhepatic RF for benign hepaticojejunostomy strictures at a mean follow-up of 7.3 months [92]. Finally, although the data are still scarce, this field merits further research, as endobiliary RFA might be added to the armamentarium of treating non-resolving benign biliary strictures. Further studies are needed to exactly assess the utility of endobiliary RFA for benign biliary strictures and to define the exact patient population for this specific treatment.

## 5. Safety and Complications

Several adverse events have been reported with the use endobiliary RFA, commonly mild self-limiting abdominal pain after the procedure, which accounts for approximately 50% of cases [93,94,95]. Additionally, bleeding was reported after percutaneous application of RFA, while post-ERCP pancreatitis was reported after endobiliary RFA. Other adverse events include hepatic artery pseudoaneurysm and hemobilia, which have been proposed to be secondary to thermal injury [31,32,33,34,35,36,37,38,39,40,41,42,43,44,45,46,47,48,49,50,51,52,53,54,55,56,57,58,59,60,61,62,63,64,65,66,67,68,69,70,71,72,73,74,75,76,77,78,79,80,81,82,83,84,85,86,87,88,89,90,91,92,93,94,95,96]. This thermal-induced adverse event can be minimized using the newest generation of RFA devices (ELRA™ catheter), which is provided with temperature control. The literature review revealed that the most common adverse event post-endobiliary RFA was mild acute cholangitis that was mostly antibiotic responsive, followed by mild self-limiting abdominal pain. Table 4 demonstrates the detailed adverse events.

## 6. Limitations and Gaps in Knowledge

Despite the emerging data and evidence in the field of endobiliary RFA, it is associated with limitations:

1. The effectiveness of endobiliary RFA is inversely correlated with tumor size and patients age, as the best performance for endobiliary RFA was for tumors ≤20 mm [97].
2. Endobiliary RFA needs to have a direct contact with the tumor, which might limit its applicability to tumors in inaccessible locations. Moreover, tumors near large blood vessels represent a therapeutic challenge [55]. Additionally, endobiliary RFA might cause a “heat-sink” effect, which leads to partial tumor destruction and cell death due to the limitation of applying maximal ablation current [98].
3. There is a scarcity of randomized controlled trials and heterogeneity in study designs, patient populations, combined therapy, RFA setting, and lack of long-term data beyond 1–2 years post-endobiliary RFA. Additionally, since the introduction of immunotherapy for CCA treatment, no studies to date have assessed the combined effect of RFA with immunotherapy, necessitating further studies to assess patient outcomes.
4. There is an inability to use RFA in pregnant patients or patients with cardiac devices or coagulation problems [99,100].

Therefore, future studies must answer these gaps by performing multicenter prospective trials and standardized treatment protocols (type of generator and electrodes, power setting, duration of ablation, single or multiple treatment sessions, and type of stent used), with uniform disease stages and longer and regular follow-up periods post-treatment.

## 7. Conclusions

Endobiliary RFA is a promising therapeutic tool that can be added to the treatment pool of biliary diseases, specifically in the palliation of malignant biliary obstruction, as the studies revealed that it extends stent patency, has a potential survival benefit, and is associated with an acceptable adverse event rate, without serious adverse events and zero mortality. Further research is warranted to refine its use and extend its indications in pancreato-biliary diseases.

## Figures and Tables

**Table 1:** Cohort uncontrolled studies of endobiliary RFA in malignant biliary obstruction.

Reference	Patients Number	Cause of MBO	Type of Study	RFA Device	Technical Success (%)	Stent Patency (Days)	Survival (Days)
Steel et al. [43]	22	CCA (*n* = 6) PC (*n* = 16)	Prospective	Habib™	95.5	85.7% at 90 d	NR
Figueroa-Barojas et al. [52]	20	CCA (*n* = 11) PC (*n* = 7) IPMN (*n* = 1) GC (*n* = 1)	Prospective	Habib™	100	100% at 30 d	NR
Dolak et al. [53]	58	MBO (causes not detailed)	Retrospective	Habib™	100	170	318
Sharaiha et al. [36]	69	CCA (*n* = 45) PC (*n* = 19) GBC (*n* = 2) GC (*n* = 1) LM (*n* = 3)	Retrospective	Habib™	100	95.65% at 30 d	343.8
Laleman et al. [54]	18	CCA (*n* = 11) PC (*n* = 7)	Prospective	ERLA™	100	110	227
Lee et al. [55]	30	CCA (*n* = 19) PC (*n* = 9) GC (*n* = 2)	Prospective	ERLA™	100	236	383

MBO: malignant biliary obstruction; CCA: cholangiocarcinoma; IPMN: intraductal papillary mucinous neoplasm; PC: pancreatic cancer; GB: gallbladder cancer; GC: gastric cancer; CC: colon cancer; LM: liver metastasis.

**Table 2:** Controlled studies of endobiliary RFA in malignant biliary obstruction.

Reference	Patient Number (RFA + Stent)	Cause of MBO (RFA + Stent)	Patient Number (Stent)	Cause of MBO (Stent)	Median Survival (Days, RFA + Stent)	Median Survival (Days, Stent)	Stent Patency (Days, RFA + Stent)	Stent Patency (Days, Stent)
Sharaiha et al. [41]	26	CCA (*n* = 18); PC (*n* = 6)	40	CCA (*n* = 19); PC (*n* = 21)	177	177	100% at 30 d	100% at 30 d
Kallis et al. [56]	23	PC (*n* = 23)	46	PC (*n* = 46)	226	123.5	472	324
Liang et al. [57]	34	CCA (*n* = 34)	42	CCA (*n* = 42)	570	480	285	250.5
Bokemayer et al. [60]	32	CCA (*n* = 32)	22	CCA (*n* = 22)	342	221	NR	NR
Yang et al. [21]	32	CCA (*n* = 32)	33	CCA (*n* = 33)	396	349	204	102
Shin et al. [58]	32	CCA (*n* = 32)	32	CCA (*n* = 32)	337	296	242	168
Kang et al. [59]	24	CCA (*n* = 18), PC (*n* = 4), and others (*n* = 2)	24	CCA (*n* = 12), PC (*n* = 10), and others (*n* = 2)	244	180	132	116

RFA: radiofrequency ablation. CCA: cholangiocarcinoma. PC: pancreatic cancer. NR: not reported in the manuscript.

**Table 3:** Endobiliary RFA for intraductal extension of ampullary tumors.

Reference	Mehendiratta et al. [76] *	Valente et al. [77] *	Suarez et al. [78] *	Rustagi et al. [79] **	Camus et al. [80] ***	Choi et al. [81] **	Tringali et al. [82] ***	Dahel et al. [83] **
Patient number	1	3	4	14	20	10	9	25
Intraductal extension, N (%)	0	2 (66.7)	NR	14 (100)	20 (100)	10 (100)	9 (100)	11 (44)
Histology of ampullary adenoma, N	LGD and HGD	LGD (2) HGD (1)	LGD (3) HGD (1)	LGD (6) HGD (6) AC (2)	LGD (15) HGD (5)	LGD (8) HGD (2)	LGD (5) HDG (3) IMC (1)	LGD (10) HGD (7) IMC (1) AC (1) NET (1)
RFA probe	Habib™	NR	Habib™	Habib™	Habib™	ELRA™	ELRA™	ELRA™
Setting		NR						
Watts	7		10	7–10	10	7	10	10
Duration (seconds)	90		60–90	90 (60–140)	30	15–90	120	120
Pre-RFA pancreatic stenting, N (%)	1 (100)	3 (100)	4 (100)	12 (85.7)	5 (25)	9 (90)	9 (100)	20 (77.5)
Post-RFA biliary stenting, N (%)	1 (100)	3 (100)	4 (100)	12 (85.7)	20 (100)	10 (100)	9 (100)	25 (100)
Technical success, N (%)	1 (100)	3 (100)	4 (100)	14 (100)	20 (100)	10 (100)	9 (100)	25 (100)
Clinical success (no recurrence)	1 (100)	3 (100)	3 (75)	12 (85.7)	14 (70)	9 (90)	6 (67)	22 (92)
Additional modalities, N (%)	0	0				0		0
APC			4 (100)	6 (42.8)	2 (10)		5 (56)	
Thermal probes			0	2 (14.3)	0		0	
PDT			0	0	0		0	
Adverse events, N (%)	0	NR	1 (25)	6 (43)	8 (40)	3 (30)	1 (11)	14 (56)
Median follow-up (months)	NR	24	2	10.9	12	9.5	21	37

* Case report. ** Retrospective. *** Prospective. LGD: low-grade dysplasia; HGD: high-grade dysplasia; AC: adenocarcinoma; IMC: intramucosal carcinoma; NET: neuroendocrine tumor; NR: not reported; RFA: radiofrequency ablation; APC: argon plasma coagulation; PDT: photodynamic therapy.

**Table 4:** Adverse events reported with endobiliary RFA for malignant biliary obstruction.

Reference	Acute Cholangitis (N)	Acute Pancreatitis (N)	Acute Cholecystitis (N)	Liver Infarction (N)	Hemobilia (N)	Abdominal Pain (N)	Others (N)
Steel et al. [43] RFA + stent (22 pts)	0	1 (4.5)	2 (9.1)	0	0	0	0
Figueroa-Barojas et al. [52] RFA + stent (20 pts)	0	1 (5)	1 (5)	0	0	0	0
Dolak et al. [53] RFA + stent (58 pts)	7	0	0	1	2	0	2
Sharaiha et al. [36] RFA + stent (69 pts)	0	1	2	0	1	3	0
Laleman et al. [54] RFA + stent (18 pts)	4	2	0	0	0	0	0
Lee et al. [55] RFA + stent (30 pts)	1	2	0	0	0	0	0
**Controlled studies**							
Sharaiha et al. [18] RFA + stent (26 pts)/stent alone (40 pts)	0/0	1/1	1/17	0/0	0/0	3/3	0/0
Kallis et al. [56] RFA + stent (23 pts)/stent alone (46 pts)	1/1	0/0	0/0	0/0	0/0	0/0	1/1
Liang et al. [57] RFA + stent (34 pts)/stent alone (42 pts)	1/2	2/1	1/0	0/0	0/0	5/7	0/0
Bokemayer et al. [60] RFA + stent (32 pts)/stent alone (22 pts)	6/NR	2/NR	0/NR	0/NR	0/NR	0/NR	2/NR
Yang et al. [21] RFA + stent (32 pts)/stent alone (33 pts)	2/1	0/1	0/0	0/0	0/1	0/0	0/0
Shin et al. [58] RFA + stent (32 pts)/stent alone (32 pts)	3/2	0/0	0/1	0/0	1/0	0/0	0/0
Kang et al. [59] RFA + stent (24 pts)/stent alone (24 pts)	1/0	0/3	0/0	0/0	0/0	0/0	0/0

Pts: patients; NR: not reported.

## Data Availability

The study materials are present with the corresponding authors and will be available upon reasonable request.

## Abbreviations

RFA	radiofrequency ablation
MBO	malignant biliary obstruction
ERCP	endoscopic retrograde cholangiopancreatography
CCA	cholangiocarcinoma
B-IPMN	Biliary intraductal papillary mucinous neoplasm
SEMS	self-expandable metal stent.

## References

[1] Green B.L., House M.G. (2019). Nonsurgical Approaches to Treat Biliary Tract and Liver Tumors. Surg. Oncol. Clin. N. Am..

[2] Buerlein R.C.D., Wang A.Y. (2019). Endoscopic Retrograde Cholangiopancreatography-Guided Ablation for Cholangiocarcinoma. Gastrointest. Endosc. Clin. N. Am..

[3] Razumilava N., Gores G.J. (2013). Classification, diagnosis, and management of cholangiocarcinoma. Clin. Gastroenterol. Hepatol..

[4] Cillo U., Fondevila C., Donadon M., Gringeri E., Mocchegiani F., Schlitt H.J., Ijzermans J.N.M., Vivarelli M., Zieniewicz K., Olde Damink S.W.M. (2019). Surgery for cholangiocarcinoma. Liver Int..

[5] Chaiteerakij R., Harmsen W.S., Marrero C.R., Aboelsoud M.M., Ndzengue A., Kaiya J., Therneau T.M., Sanchez W., Gores G.J., Roberts L.R. (2014). A new clinically based staging system for perihilar cholangiocarcinoma. Am. J. Gastroenterol..

[6] Nelson J.W., Ghafoori A.P., Willett C.G., Tyler D.S., Pappas T.N., Clary B.M., Hurwitz H.I., Bendell J.C., Morse M.A., Clough R.W. (2009). Concurrent chemoradiotherapy in resected extrahepatic cholangiocarcinoma. Int. J. Radiat. Oncol. Biol. Phys..

[7] Jung J.H., Lee H.J., Lee H.S., Jo J.H., Cho I.R., Chung M.J., Park J.Y., Park S.W., Song S.Y., Bang S. (2017). Benefit of neoadjuvant concurrent chemoradiotherapy for locally advanced perihilar cholangiocarcinoma. World J. Gastroenterol..

[8] Jarosova J., Macinga P., Hujova A., Kral J., Urban O., Spicak J., Hucl T. (2021). Endoscopic radiofrequency ablation for malignant biliary obstruction. World J. Gastrointest. Oncol..

[9] Tarek M. (2005). Membrane electroporation: A molecular dynamics simulation. Biophys. J..

[10] Zhang K., Yu J., Yu X., Han Z., Cheng Z., Liu F., Liang P. (2018). Clinical and survival outcomes of percutaneous microwave ablation for intrahepatic cholangiocarcinoma. Int. J. Hyperth..

[11] Yu M.A., Liang P., Yu X.L., Cheng Z.G., Han Z.Y., Liu F.Y., Yu J. (2011). Sonography-guided percutaneous microwave ablation of intrahepatic primary cholangiocarcinoma. Eur. J. Radiol..

[12] Glazer D.I., Tatli S., Shyn P.B., Vangel M.G., Tuncali K., Silverman S.G. (2017). Percutaneous Image-Guided Cryoablation of Hepatic Tumors: Single-Center Experience with Intermediate to Long-Term Outcomes. AJR Am. J. Roentgenol..

[13] Dougherty T.J., Gomer C.J., Henderson B.W., Jori G., Kessel D., Korbelik M., Moan J., Peng Q. (1998). Photodynamic therapy. J. Natl. Cancer Inst..

[14] Cheon Y.K., Cho Y.D., Baek S.H., Cha S.W., Moon J.H., Kim Y.S., Lee J.S., Lee M.S., Shim C.S., Kim B.S. (2004). Comparison of survival of advanced hilar cholangiocarcinoma after biliary drainage alone versus photodynamic therapy with external drainage. Korean J. Gastroenterol..

[15] Zoepf T., Jakobs R., Arnold J.C., Apel D., Riemann J.F. (2005). Palliation of nonresectable bile duct cancer: Improved survival after photodynamic therapy. Am. J. Gastroenterol..

[16] Ortner M.E., Caca K., Berr F., Liebetruth J., Mansmann U., Huster D., Voderholzer W., Schachschal G., Mossner J., Lochs H. (2003). Successful photodynamic therapy for nonresectable cholangiocarcinoma: A randomized prospective study. Gastroenterology.

[17] Labib P.L., Davidson B.R., Sharma R.A., Pereira S.P. (2017). Locoregional therapies in cholangiocarcinoma. Hepat. Oncol..

[18] Nault J.C., Sutter O., Nahon P., Ganne-Carrie N., Seror O. (2018). Percutaneous treatment of hepatocellular carcinoma: State of the art and innovations. J. Hepatol..

[19] Chahal P., Baron T.H. (2006). Endoscopic palliation of cholangiocarcinoma. Curr. Opin. Gastroenterol..

[20] Sofi A.A., Khan M.A., Das A., Sachdev M., Khuder S., Nawras A., Lee W. (2018). Radiofrequency ablation combined with biliary stent placement versus stent placement alone for malignant biliary strictures: A systematic review and meta-analysis. Gastrointest. Endosc..

[21] Yang J., Wang J., Zhou H., Zhou Y., Wang Y., Jin H., Lou Q., Zhang X. (2018). Efficacy and safety of endoscopic radiofrequency ablation for unresectable extrahepatic cholangiocarcinoma: A randomized trial. Endoscopy.

[22] Wu J., Zhou Z., Huang Y., Deng X., Zheng S., He S., Huang G., Hu B., Shi M., Liao W. (2024). Radiofrequency ablation: Mechanisms and clinical applications. MedComm.

[23] Goldberg S.N., Gazelle G.S., Mueller P.R. (2000). Thermal ablation therapy for focal malignancy: A unified approach to underlying principles, techniques, and diagnostic imaging guidance. AJR Am. J. Roentgenol..

[24] Higgins H., Berger D.L. (2006). RFA for liver tumors: Does it really work?. Oncologist.

[25] Ishikawa T., Kubota T., Horigome R., Kimura N., Honda H., Iwanaga A., Seki K., Honma T., Yoshida T. (2013). Radiofrequency ablation during continuous saline infusion can extend ablation margins. World J. Gastroenterol..

[26] Izzo F., Granata V., Grassi R., Fusco R., Palaia R., Delrio P., Carrafiello G., Azoulay D., Petrillo A., Curley S.A. (2019). Radiofrequency Ablation and Microwave Ablation in Liver Tumors: An Update. Oncologist.

[27] Curley S.A. (2001). Radiofrequency ablation of malignant liver tumors. Oncologist.

[28] Hanna N.N. (2004). Radiofrequency ablation of primary and metastatic hepatic malignancies. Clin. Color. Cancer.

[29] Regier M., Chun F. (2015). Thermal Ablation of Renal Tumors: Indications, Techniques and Results. Dtsch. Arztebl. Int..

[30] Goldberg S.N., Gazelle G.S. (2001). Radiofrequency tissue ablation: Physical principles and techniques for increasing coagulation necrosis. Hepatogastroenterology.

[31] Tal A.O., Vermehren J., Friedrich-Rust M., Bojunga J., Sarrazin C., Zeuzem S., Trojan J., Albert J.G. (2014). Intraductal endoscopic radiofrequency ablation for the treatment of hilar non-resectable malignant bile duct obstruction. World J. Gastrointest. Endosc..

[32] Monga A., Gupta R., Ramchandani M., Rao G.V., Santosh D., Reddy D.N. (2011). Endoscopic radiofrequency ablation of cholangiocarcinoma: New palliative treatment modality (with videos). Gastrointest. Endosc..

[33] Alitti C., Rode A., Trillaud H., Merle P., Blanc J.F., Blaise L., Demory A., Nkontchou G., Grando V., Ziol M. (2024). Long-term oncological results of percutaneous radiofrequency ablation for intrahepatic cholangiocarcinoma. Liver Int..

[34] Chu H.H., Kim J.H., Shin Y.M., Won H.J., Kim P.N. (2021). Percutaneous Radiofrequency Ablation for Recurrent Intrahepatic Cholangiocarcinoma After Curative Resection: Multivariable Analysis of Factors Predicting Survival Outcomes. AJR Am. J. Roentgenol..

[35] Xiang X., Hu D., Jin Z., Liu P., Lin H. (2020). Radiofrequency Ablation vs. Surgical Resection for Small Early-Stage Primary Intrahepatic Cholangiocarcinoma. Front. Oncol..

[36] Sharaiha R.Z., Sethi A., Weaver K.R., Gonda T.A., Shah R.J., Fukami N., Kedia P., Kumta N.A., Clavo C.M., Saunders M.D. (2015). Impact of Radiofrequency Ablation on Malignant Biliary Strictures: Results of a Collaborative Registry. Dig. Dis. Sci..

[37] Dumonceau J.M., Tringali A., Papanikolaou I.S., Blero D., Mangiavillano B., Schmidt A., Vanbiervliet G., Costamagna G., Deviere J., Garcia-Cano J. (2018). Endoscopic biliary stenting: Indications, choice of stents, and results: European Society of Gastrointestinal Endoscopy (ESGE) Clinical Guideline—Updated October 2017. Endoscopy.

[38] Cho J.H., Jeong S., Kim E.J., Kim J.M., Kim Y.S., Lee D.H. (2018). Long-term results of temperature-controlled endobiliary radiofrequency ablation in a normal swine model. Gastrointest. Endosc..

[39] Kim E.J., Chung D.H., Kim Y.J., Kim Y.S., Park Y.H., Kim K.K., Cho J.H. (2018). Endobiliary radiofrequency ablation for distal extrahepatic cholangiocarcinoma: A clinicopathological study. PLoS ONE.

[40] Li T.F., Huang G.H., Li Z., Hao C.F., Ren J.Z., Duan X.H., Zhang K., Chen C., Han X.W., Jiao D.C. (2015). Percutaneous transhepatic cholangiography and intraductal radiofrequency ablation combined with biliary stent placement for malignant biliary obstruction. J. Vasc. Interv. Radiol..

[41] Sharaiha R.Z., Natov N., Glockenberg K.S., Widmer J., Gaidhane M., Kahaleh M. (2014). Comparison of metal stenting with radiofrequency ablation versus stenting alone for treating malignant biliary strictures: Is there an added benefit?. Dig. Dis. Sci..

[42] Wu T.T., Li H.C., Li W.M., Ao G.K., Lin H., Zheng F., Song J.Y. (2015). Percutaneous Intraluminal Radiofrequency Ablation for Malignant Extrahepatic Biliary Obstruction: A Safe and Feasible Method. Dig. Dis. Sci..

[43] Steel A.W., Postgate A.J., Khorsandi S., Nicholls J., Jiao L., Vlavianos P., Habib N., Westaby D. (2011). Endoscopically applied radiofrequency ablation appears to be safe in the treatment of malignant biliary obstruction. Gastrointest. Endosc..

[44] Barret M., Leblanc S., Vienne A., Rouquette A., Beuvon F., Chaussade S., Prat F. (2015). Optimization of the generator settings for endobiliary radiofrequency ablation. World J. Gastrointest. Endosc..

[45] Xia M., Qin W., Hu B. (2023). Endobiliary radiofrequency ablation for unresectable malignant biliary strictures: Survival benefit perspective. Dig. Endosc..

[46] Coucke E.M., Akbar H., Kahloon A., Lopez P.P. (2025). Biliary Obstruction. StatPearls.

[47] Shaib Y., El-Serag H.B. (2004). The epidemiology of cholangiocarcinoma. Semin. Liver Dis..

[48] Ferlay J., Shin H.R., Bray F., Forman D., Mathers C., Parkin D.M. (2010). Estimates of worldwide burden of cancer in 2008: GLOBOCAN 2008. Int. J. Cancer.

[49] Qureshi K., Jesudoss R., Al-Osaimi A.M. (2014). The treatment of cholangiocarcinoma: A hepatologist’s perspective. Curr. Gastroenterol. Rep..

[50] Chu K.J., Kawaguchi Y., Wang H., Jiang X.Q., Hasegawa K. (2024). Update on the Diagnosis and Treatment of Combined Hepatocellular Cholangiocarcinoma. J. Clin. Transl. Hepatol..

[51] Wu T.T., Li W.M., Li H.C., Ao G.K., Zheng F., Lin H. (2017). Percutaneous Intraductal Radiofrequency Ablation for Extrahepatic Distal Cholangiocarcinoma: A Method for Prolonging Stent Patency and Achieving Better Functional Status and Quality of Life. Cardiovasc. Interv. Radiol..

[52] Figueroa-Barojas P., Bakhru M.R., Habib N.A., Ellen K., Millman J., Jamal-Kabani A., Gaidhane M., Kahaleh M. (2013). Safety and efficacy of radiofrequency ablation in the management of unresectable bile duct and pancreatic cancer: A novel palliation technique. J. Oncol..

[53] Dolak W., Schreiber F., Schwaighofer H., Gschwantler M., Plieschnegger W., Ziachehabi A., Mayer A., Kramer L., Kopecky A., Schrutka-Kolbl C. (2014). Endoscopic radiofrequency ablation for malignant biliary obstruction: A nationwide retrospective study of 84 consecutive applications. Surg. Endosc..

[54] Laleman W., van der Merwe S., Verbeke L., Vanbeckevoort D., Aerts R., Prenen H., Van Cutsem E., Verslype C. (2017). A new intraductal radiofrequency ablation device for inoperable biliopancreatic tumors complicated by obstructive jaundice: The IGNITE-1 study. Endoscopy.

[55] Lee Y.N., Jeong S., Choi H.J., Cho J.H., Cheon Y.K., Park S.W., Kim Y.S., Lee D.H., Moon J.H. (2019). The safety of newly developed automatic temperature-controlled endobiliary radiofrequency ablation system for malignant biliary strictures: A prospective multicenter study. J. Gastroenterol. Hepatol..

[56] Kallis Y., Phillips N., Steel A., Kaltsidis H., Vlavianos P., Habib N., Westaby D. (2015). Analysis of Endoscopic Radiofrequency Ablation of Biliary Malignant Strictures in Pancreatic Cancer Suggests Potential Survival Benefit. Dig. Dis. Sci..

[57] Liang H., Peng Z., Cao L., Qian S., Shao Z. (2015). Metal Stenting with or without Endobiliary Radiofrequency Ablation for Unresectable Extrahepatic Cholangiocarcinoma. J. Cancer Ther..

[58] Shin I.S., Moon J.H., Lee Y.N., Myeong J.H., Lee T.H., Yang J.K., Cho Y.D., Park S.H. (2024). Impact of temperature-controlled endobiliary radiofrequency ablation for inoperable hilar cholangiocarcinoma: A propensity score-matched analysis. Endosc. Int. Open.

[59] Kang H., Chung M.J., Cho I.R., Jo J.H., Lee H.S., Park J.Y., Park S.W., Song S.Y., Bang S. (2021). Efficacy and safety of palliative endobiliary radiofrequency ablation using a novel temperature-controlled catheter for malignant biliary stricture: A single-center prospective randomized phase II TRIAL. Surg. Endosc..

[60] Bokemeyer A., Matern P., Bettenworth D., Cordes F., Nowacki T.M., Heinzow H., Kabar I., Schmidt H., Ullerich H., Lenze F. (2019). Endoscopic Radiofrequency Ablation Prolongs Survival of Patients with Unresectable Hilar Cholangiocellular Carcinoma—A Case-Control Study. Sci. Rep..

[61] Nakajima T., Kondo Y., Miyazaki M., Okui K. (1988). A histopathologic study of 102 cases of intrahepatic cholangiocarcinoma: Histologic classification and modes of spreading. Hum. Pathol..

[62] Ritchie D.J., Okamoto K., White S.L. (2019). Intraductal papillary mucinous neoplasm of the biliary tract: A precursor lesion to cholangiocarcinoma. Radiol. Case Rep..

[63] Rocha F.G., Lee H., Katabi N., DeMatteo R.P., Fong Y., D’Angelica M.I., Allen P.J., Klimstra D.S., Jarnagin W.R. (2012). Intraductal papillary neoplasm of the bile duct: A biliary equivalent to intraductal papillary mucinous neoplasm of the pancreas?. Hepatology.

[64] Lee S.S., Kim M.H., Lee S.K., Jang S.J., Song M.H., Kim K.P., Kim H.J., Seo D.W., Song D.E., Yu E. (2004). Clinicopathologic review of 58 patients with biliary papillomatosis. Cancer.

[65] Yeung Y.P., AhChong K., Chung C.K., Chun A.Y. (2003). Biliary papillomatosis: Report of seven cases and review of English literature. J. Hepatobiliary Pancreat. Surg..

[66] Natov N.S., Horton L.C., Hegde S.R. (2017). Successful endoscopic treatment of an intraductal papillary neoplasm of the bile duct. World J. Gastrointest. Endosc..

[67] Delaney S., Zhou Y., Pawa S., Pawa R. (2021). Intraductal papillary neoplasm of the left hepatic duct treated with endoscopic retrograde cholangiopancreatography guided radiofrequency ablation. Clin. J. Gastroenterol..

[68] Tang W., Qiu J.G., Wei X.F., Xiao H., Deng X., Wang S.D., Du C.Y., Wu Q. (2021). Endoscopic Endoluminal Radiofrequency Ablation and Single-Operator Peroral Cholangioscopy System (SpyGlass) in the Diagnosis and Treatment of Intraductal Papillary Neoplasm of the Bile Duct: A Case Report and Literature Review. Front. Med..

[69] Chathadi K.V., Khashab M.A., Acosta R.D., Chandrasekhara V., Eloubeidi M.A., Faulx A.L., Fonkalsrud L., Lightdale J.R., Salztman J.R., ASGE Standards of Practice Committee (2015). The role of endoscopy in ampullary and duodenal adenomas. Gastrointest. Endosc..

[70] Kang S.H., Kim K.H., Kim T.N., Jung M.K., Cho C.M., Cho K.B., Han J.M., Kim H.G., Kim H.S. (2017). Therapeutic outcomes of endoscopic papillectomy for ampullary neoplasms: Retrospective analysis of a multicenter study. BMC Gastroenterol..

[71] Haraldsson E., Swahn F., Verbeke C., Mattsson J.S., Enochsson L., Ung K.A., Lundell L., Heuchel R., Lohr J.M., Arnelo U. (2015). Endoscopic papillectomy and KRAS expression in the treatment of adenoma in the major duodenal papilla. Scand. J. Gastroenterol..

[72] Bohnacker S., Seitz U., Nguyen D., Thonke F., Seewald S., deWeerth A., Ponnudurai R., Omar S., Soehendra N. (2005). Endoscopic resection of benign tumors of the duodenal papilla without and with intraductal growth. Gastrointest. Endosc..

[73] Ceppa E.P., Burbridge R.A., Rialon K.L., Omotosho P.A., Emick D., Jowell P.S., Branch M.S., Pappas T.N. (2013). Endoscopic versus surgical ampullectomy: An algorithm to treat disease of the ampulla of Vater. Ann. Surg..

[74] Jordan P.H., Ayala G., Rosenberg W.R., Kinner B.M. (2002). Treatment of ampullary villous adenomas that may harbor carcinoma. J. Gastrointest. Surg..

[75] Tran T.C., Vitale G.C. (2004). Ampullary tumors: Endoscopic versus operative management. Surg. Innov..

[76] Mehendiratta V., Desilets D.J. (2015). Use of radiofrequency ablation probe for eradication of residual adenoma after ampullectomy. Gastrointest. Endosc..

[77] Valente R., Urban O., Del Chiaro M., Capurso G., Blomberg J., Lohr J.M., Arnelo U. (2015). ERCP-directed radiofrequency ablation of ampullary adenomas: A knife-sparing alternative in patients unfit for surgery. Endoscopy.

[78] Suarez A.L., Cote G.A., Elmunzer B.J. (2016). Adjunctive radiofrequency ablation for the endoscopic treatment of ampullary lesions with intraductal extension (with video). Endosc. Int. Open.

[79] Rustagi T., Irani S., Reddy D.N., Abu Dayyeh B.K., Baron T.H., Gostout C.J., Levy M.J., Martin J., Petersen B.T., Ross A. (2017). Radiofrequency ablation for intraductal extension of ampullary neoplasms. Gastrointest. Endosc..

[80] Camus M., Napoleon B., Vienne A., Le Rhun M., Leblanc S., Barret M., Chaussade S., Robin F., Kaddour N., Prat F. (2018). Efficacy and safety of endobiliary radiofrequency ablation for the eradication of residual neoplasia after endoscopic papillectomy: A multicenter prospective study. Gastrointest. Endosc..

[81] Choi Y.H., Yoon S.B., Chang J.H., Lee I.S. (2021). The Safety of Radiofrequency Ablation Using a Novel Temperature-Controlled Probe for the Treatment of Residual Intraductal Lesions after Endoscopic Papillectomy. Gut Liver.

[82] Tringali A., Matteo M.V., Orlandini B., Barbaro F., Perri V., Zhang Q., Ricci R., Costamagna G. (2021). Radiofrequency ablation for intraductal extension of ampullary adenomatous lesions: Proposal for a standardized protocol. Endosc. Int. Open.

[83] Dahel Y., Caillol F., Ratone J.P., Zemmour C., Palen A., Garnier J., Ewald J., Turrini O., Hoibian S., Giovannini M. (2025). Safety of intrabiliary radiofrequency ablation in cases of residual and recurrent neoplasia after endoscopic papillectomy. Endosc. Int. Open.

[84] Kadayifci A., Atar M., Forcione D.G., Casey B.W., Kelsey P.B., Brugge W.R. (2016). Radiofrequency ablation for the management of occluded biliary metal stents. Endoscopy.

[85] Nayar M.K., Oppong K.W., Bekkali N.L.H., Leeds J.S. (2018). Novel temperature-controlled RFA probe for treatment of blocked metal biliary stents in patients with pancreaticobiliary cancers: Initial experience. Endosc. Int. Open.

[86] Ma M.X., Jayasekeran V., Chong A.K. (2019). Benign biliary strictures: Prevalence, impact, and management strategies. Clin. Exp. Gastroenterol..

[87] Wong M.Y., Saxena P., Kaffes A.J. (2020). Benign Biliary Strictures: A Systematic Review on Endoscopic Treatment Options. Diagnostics.

[88] Obuch J.C., Wagh M.S. (2017). Endoscopic therapy for benign biliary strictures: Evaluation of metal vs. plastic biliary stents. Hepatobiliary Surg. Nutr..

[89] Ni D.J., Yang Q.F., Nie L., Xu J., He S.Z., Yao J. (2024). The past, present, and future of endoscopic management for biliary strictures: Technological innovations and stent advancements. Front. Med..

[90] Mallette K., Hawel J., Elnahas A., Alkhamesi N.A., Schlachta C.M., Tang E.S. (2023). The utility of self-expanding metal stents in benign biliary strictures—A retrospective case series. BMC Gastroenterol..

[91] Hu B., Gao D.J., Wu J., Wang T.T., Yang X.M., Ye X. (2014). Intraductal radiofrequency ablation for refractory benign biliary stricture: Pilot feasibility study. Dig. Endosc..

[92] Ozdemir M., Kucukay F., Ozdemir F.A.E., Acu R., Tola M., Yurdakul M. (2018). Percutaneous endobiliary radiofrequency ablation for refractory benign hepaticojejunostomy and biliary strictures. Diagn. Interv. Imaging.

[93] Cha B.H., Jang M.J., Lee S.H. (2021). Survival Benefit of Intraductal Radiofrequency Ablation for Malignant Biliary Obstruction: A Systematic Review with Meta-Analysis. Clin. Endosc..

[94] de Jong D.M., Fritzsche J.A., Audhoe A.S., Yi S.S.L., Bruno M.J., Voermans R.P., van Driel L. (2022). Comparison of Intraductal RFA Plus Stent versus Stent-Only Treatment for Unresectable Perihilar Cholangiocarcinoma—A Systematic Review and Meta-Analysis. Cancers.

[95] Zheng X., Bo Z.Y., Wan W., Wu Y.C., Wang T.T., Wu J., Gao D.J., Hu B. (2016). Endoscopic radiofrequency ablation may be preferable in the management of malignant biliary obstruction: A systematic review and meta-analysis. J. Dig. Dis..

[96] Laquiere A., Boustiere C., Leblanc S., Penaranda G., Desilets E., Prat F. (2016). Safety and feasibility of endoscopic biliary radiofrequency ablation treatment of extrahepatic cholangiocarcinoma. Surg. Endosc..

[97] Jiang Y.Q., Wang Z.X., Deng Y.N., Yang Y., Wang G.Y., Chen G.H. (2019). Efficacy of Hepatic Resection vs. Radiofrequency Ablation for Patients with Very-Early-Stage or Early-Stage Hepatocellular Carcinoma: A Population-Based Study with Stratification by Age and Tumor Size. Front. Oncol..

[98] Weber J.C., Navarra G., Jiao L.R., Nicholls J.P., Jensen S.L., Habib N.A. (2002). New technique for liver resection using heat coagulative necrosis. Ann. Surg..

[99] Koda M., Ueki M., Maeda Y., Mimura K.I., Okamoto K., Matsunaga Y., Kawakami M., Hosho K., Murawaki Y. (2004). The influence on liver parenchymal function and complications of radiofrequency ablation or the combination with transcatheter arterial embolization for hepatocellular carcinoma. Hepatol. Res..

[100] Livraghi T., Meloni F., Di Stasi M., Rolle E., Solbiati L., Tinelli C., Rossi S. (2008). Sustained complete response and complications rates after radiofrequency ablation of very early hepatocellular carcinoma in cirrhosis: Is resection still the treatment of choice?. Hepatology.

